# Supplementary figures and images for: A flexible framework for multi-particle refinement in cryo-electron tomography
Source: PLoS Biol. 2021 Aug 26;19(8):e3001319. doi: 10.1371/journal.pbio.3001319 (PMC8389456; doi:10.1371/journal.pbio.3001319)

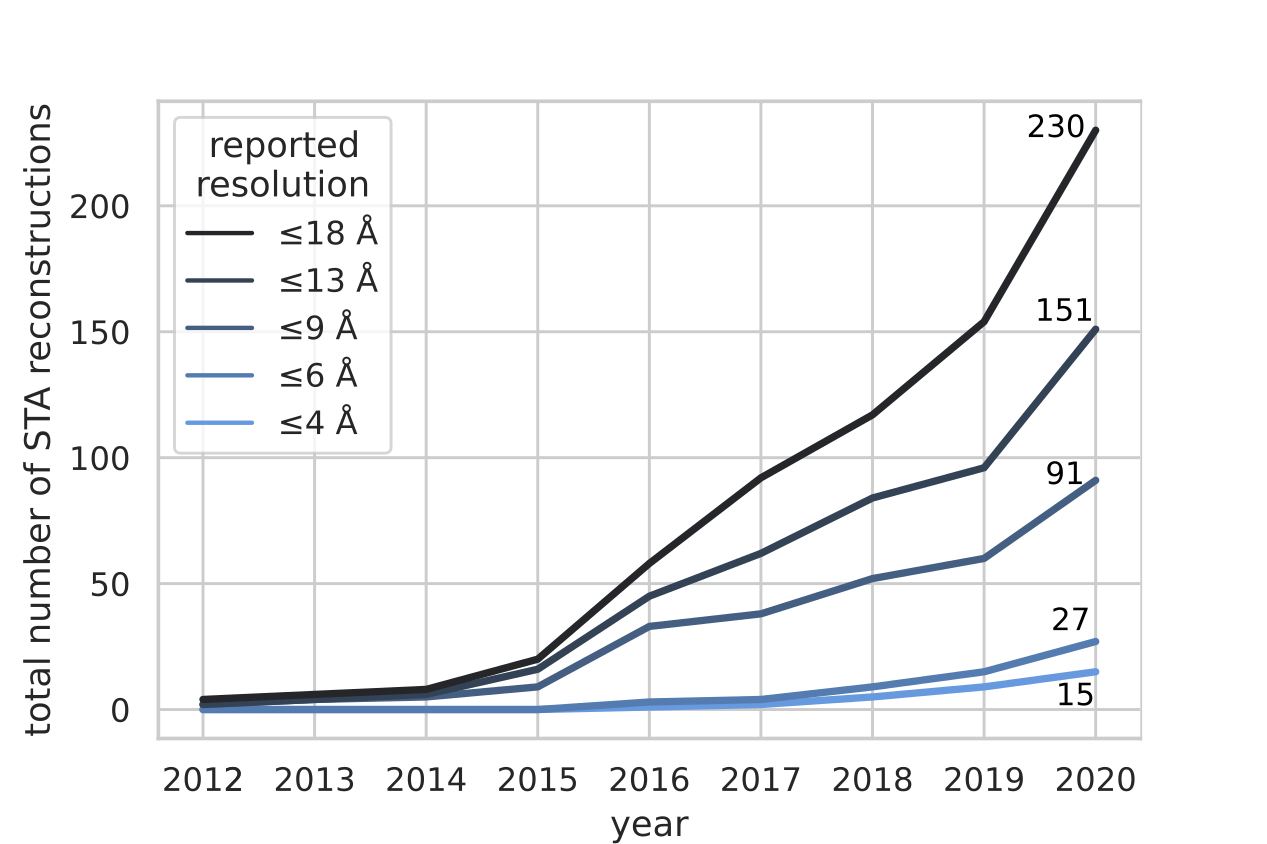

Supplement: S1 Fig — The data used to produce this figure can be found at https://www.ebi.ac.uk/pdbe/emdb/statistics_main.html. EMDB, Electron Microscopy Data Bank; STA, subtomogram averaging. (TIF) [file pbio.3001319.s001.tif]

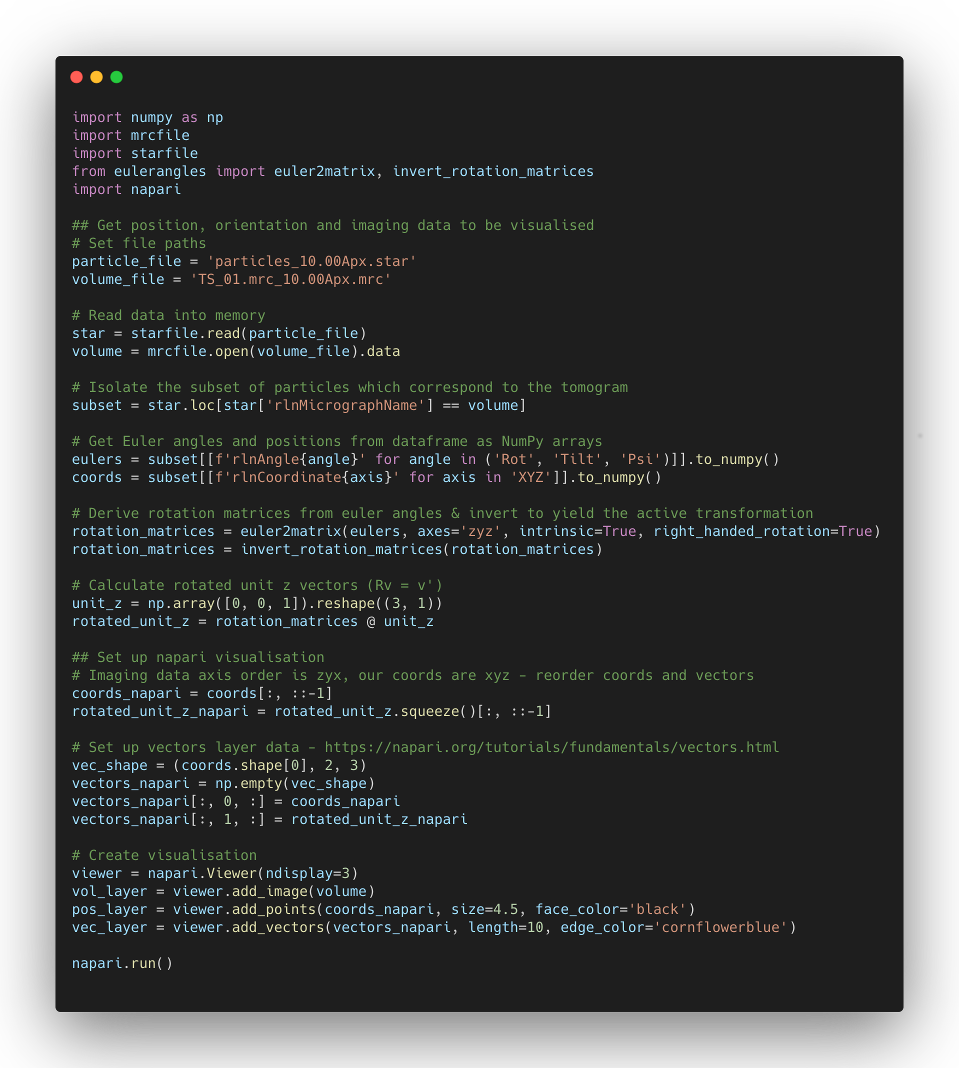

Supplement: S2 Fig — (TIF) [file pbio.3001319.s002.tif]
