# Supplementary material for: A flexible framework for multi-particle refinement in cryo-electron tomography
Source: PLoS Biol. 2021 Aug 26;19(8):e3001319. doi: 10.1371/journal.pbio.3001319 (PMC8389456; doi:10.1371/journal.pbio.3001319)
Supplement: Attachment — Submitted filename: point-by-point-response.pdf [file pbio.3001319.s003.pdf]

Dear Dr. Hodge,

Firstly, we would like to thank you for your interest in our work and for the smooth, efficient handling of the editorial process.

Given that to satisfy the reviewers's comments the manuscript has undergone a major restructuring, we cannot provide a 'track-changes' version but highlight the most important additions in yellow.
We also considered your suggestion to modify the title into 'Streamlined pipeline to facilitate high-resolution subtomogram averaging'. Our work providing a general framework rather than a pipeline, and considering the reviewers' comments on our coupling between geometrical approaches and mutli-particle refinement, we propose a modified title 'A framework for ab initio and geometrical approaches to multi-particle refinement in cryo-electron tomography' that should hopefully satisfy all parties.
As you requested, we now provide quantitative information underlying the data summarised in the figures and results of our paper at the zenodo open-access repository, add the corresponding doi to the data availability statement and provide the links to the data in the figure fegends.

Secondly, we would like to thank the reviewers for their recognition of the value provided by our work. The individual comments are addressed in the following point-by-point response.

Reviewer 1
In this manuscript, the authors describe an integrated workflow for subtomogram averaging by combining Dynamo with the recently published Warp-RELION-M pipeline. The workflow allows simplified metadata handling, increased automation, and flexible procedure. Moreover, the authors establish an open and collaborative online platform for sharing cryo-ET data analysis knowledge. The authors identify the problem that, while subtomogram averaging processing has developed alongside single particle analysis and is becoming increasingly more powerful, the software technology is fragmented, and users often have to become creative in choosing how they want to process and analyze data. This makes it difficult particularly for newcomers to cryo-ET.
Overall, this work will be very useful to the field of cryo-ET. Seeing all the different customization tools in Dynamo combined with the powerful refinement of Warp-RELION-M is really exciting. The online platform is a great addition that will hopefully help increase communication among users in the field and allow continued improvement of the methods.

> Lines 106-148: How does the accuracy of Dynamo fiducial tracking and tilt series alignment compare with that was done with IMOD alone?

We originally chose to use Dynamo to generate the fiducial positions because of issues with batchruntomo for our minicell dataset. Specifically, the initial bead model would not track well because the beads were often in a plane quite a distance from the plane of alignment. This problem was addressed in IMOD 4.10.35, but meant that batchruntomo was not a most suitable solution for alignment of these data at the time the work was completed.

From the 5 tilt-series subset of EMPIAR-10164 we obtained a 3.8 Å reconstruction prior to refinement of the tilt-series geometry in *M*. This is extremely similar to the 3.9 Å reconstruction from the same dataset in Turonova *et al.* 2017 which made use of IMOD for tilt-series alignment. The similar values indicate that the alignment accuracy can be broadly similar for optimal datasets.

> Lines 185-205: The authors highlight an important point: metadata handling can be a pain. Aside from a nice visualization of particle poses in Napari, I wonder if there is anything else they can show in this results section to guide the user (especially a newcomer) through the use of the different packages that they discuss. For example, a flowchart to go with the source code provided.

An excellent suggestion, a flowchart has been added to complement the source code and visualisation.

> Lines 240-242: Some clarity or description of how these geometric orientations were generated in Dynamo would be helpful. For example, for figure 3a I assume that the virus membrane was segmented, and particles were oriented orthogonal to the membrane surface. However, in 3b it is unclear to me how those orientations were produced.

Additional descriptions have been added to the legend for Figure 3.

> Lines 293-324: It will be very beneficial to show a flowchart of the detailed processes used, along with stepwise results shown, so the readers can have a better understanding and expectation of what to produce while trying the workflow on the VLPs or similar datasets.

We chose to outline the general procedure in Figure 1 and provide a detailed, step-by-step walkthrough with all intermediate processing steps and small scripts used for the HIV dataset at https://teamtomo.org/walkthroughs/EMPIAR-10164/introduction.html. We have made the existence of this guide significantly more clear in the manuscript and hope this satisfies the reviewers request.

> Line 299: What's the method for CTF correction in this proposed workflow, and how does it compare to NovaCTF used in the referenced VLP STA paper?

The CTF estimation and correction procedure in the proposed framework is that implemented in Warp. It is similar to NovaCTF in that they both correct according to a model which accounts for both the defocus gradient due to tilt and the defocus gradient due to sample thickness.

In the NovaCTF workflow, tomograms are typically reconstructed and subvolumes are extracted from these tomograms. In Warp, particles are reconstructed directly from the tilt-series one by one. NovaCTF is an efficient implementation of defocus-gradient corrected back-projection (Jensen and Kornberg 2000), dividing the volume into a series of slabs, each with a correction applied, which is correct (according to the 3D CTF model) at the z-center of the slab. Particles centered above and below the z-center of each reconstructed slab will have small errors in their defocus with respect to the 3D ctf model because of this. Warp reconstructs arbitrary regions of interest with the correct (according to the 3D CTF model) defocus at the center of the region of interest. In practice, the small errors in defocus due to slab thickness are unlikely to become limiting at the resolutions commonly achieved when working with tilt-series data.

> Lines 305-306: The alignment parameters (angular search, steps, etc.) should be at least briefly mentioned here. Same for the lines 314-316.

We have added details about the angular search parameters at the specified lines. We shy away from referring to the angular step here because the two programs used for rigid-body refinements (RELION and Dynamo) differ greatly in how these parameters are used within a refinement. In Dynamo, the angular step specified defines the first level of a multi-level grid (https://wiki.dynamo.biozentrum.unibas.ch/w/index.php/Multilevel_refinement). In RELION, the angular search is adapted according to the current resolution estimate. In our experience, and the experience of others (https://groups.google.com/g/warp-em/c/eV5xrn2GllM/m/3cpdriI3BAAJ), this adaptive procedure is not working as expected when perofrming subtomogram averaging in RELION 3.X.

> Lines 308-316 If an average was produced in Dynamo with 19,000 - 26,000 particles, why is it also beneficial to do more refinement in RELION? The authors should emphasize why using RELION is better than using only Dynamo. This could be done potentially by comparing resolutions of final averages without using RELION and only using Dynamo, Warp, and M. A clear explanation of the advantages of RELION over Dynamo for these steps will be helpful.

Warp is designed to use RELION as a subtomogram averaging 'engine' and as such generates representations of the Fourier space sampling of each particle which work in RELION. Whilst it is possible to format this data for use in Dynamo, this would require another conversion step and is

not implemented as part of this work. We recommend to use RELION if it works for your project as it benefits directly from the knowledge of Fourier space sampling provided by Warp, simplifying the experience for the user. Initial subtomogram alignments were performed in Dynamo because particle picking was performed in that ecosystem and tools for visualising particle positions and orientations are readily available within Dynamo, allowing for quick analysis of internal consistency after a subtomogram alignment experiment. Some explanation has been added to the 'Interfacing Dynamo and the Warp-RELION-M pipeline' section for clarity. More generally

> Lines 359-363: Why were particles extracted in Dynamo for an initial template, and then further extracted from Warp and brought into Dynamo?

This workflow removes an unnecessary metadata conversion step, allowing particles to be directly extracted from tomograms reconstructed at a large pixel size in Warp and proceeding directly to initial model generation in Dynamo. To work at smaller pixel sizes it becomes necessary to reconstruct the particles at a smaller pixel size in Warp.

> Lines 361-375: Again, similar to the comment #4 above, showing the workflow and intermediate results in the form of a flowchart would be very beneficial for new users to learn/try this method on similar data.

See response to comment #4 above.

> Line 373-375: What exactly were attempted to disentangle structural heterogeneity and how did they fail?

Info added in paper, briefly - multireference classification in Dynamo and maximum likelihood multireference classification in RELION in a variety of masks. One of the failure modes appears to be higher-order chemosensory array structure biasing the classification. The following figure shows a failed classification experiment (performed in RELION) on the core-signalling unit which appears to classify according to directions along which the higher-order chemosensory array structure is reinforced constructively in projection images.

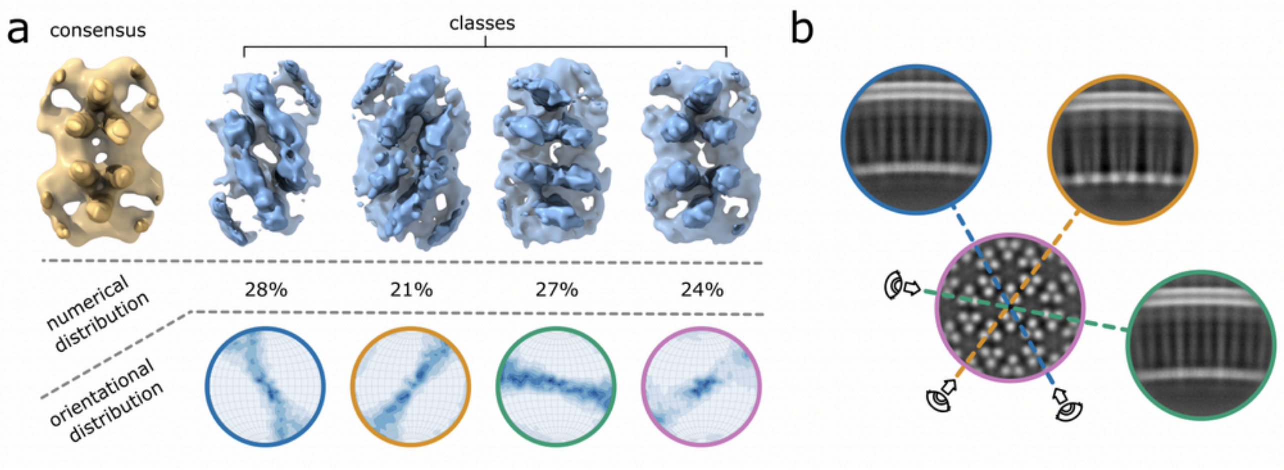

We chose to avoid labouring this point as we believe it is a problem specific to our data rather than a general limitation of multireference classification in RELION or Dynamo. Another failure mode specific to RELION was an even division of the dataset into identical classes, this was observed for a variety of initial low-pass values and many values of the tau fudge factor which modifies the relative weights of the prior and the data.

> At the end, a detailed section on hardware requirements and how to connect the systems/computers together (i.e., the need for Linux vs. Windows operation systems, CPU/GPU requirements, how to properly connect these programs to one another locally or over the network,

etc.) would be critical for new users to set the system up.

This information is provided in the guide on teamtomo.org
https://teamtomo.org/walkthroughs/EMPIAR-10164/introduction.html#requirements-and-setup
We have added some notes about our network setup.

> A note on computing time took for different steps in the workflow of the reported two example projects, with information of what computation power were used, would be beneficial for new users to plan for setting up the system.

This information is provided in the guide
https://teamtomo.org/walkthroughs/EMPIAR-10164/introduction.html
for the HIV dataset.

Reviewer 2
Summary: Burt et al describe new software packages facilitating data transfer between Dynamo, M, Relion, and Warp and increased automation. Use of these tools is illustrated via application to EMPIAR-10164 and an E. Coli chemosensory array. Increased integration between these packages and the associated flexibility will certainly be appreciated by users. Generally the paper is clear and well-written. If I were to restrict myself to a single, high-level comment, it would be that more explicit, tutorial examples would be helpful, but perhaps the authors have choosen to provide that elsewhere. See below for more specific comments.

Indeed, we choose to provide a complete tutorial as a 'living document' which we are free to modify at a later date https://teamtomo.org/walkthroughs/EMPIAR-10164/introduction.html, acknowledging that best practices are very much in flux and this workflow will likely need to be modified as the software packages it includes are updated moving forwards.

1) Lines 84-86: I suggest being more explicit about the facts that the final stages of "modern" STA refinement has more in common with SPA than with classical SPA, and that tilt series alignments and subvolumes are no longer considered fixed , but rather as initial estimates subject to further optimization.

Thank you for the suggestion, we have made this more clear in the text.

2) Lines 109-119: IMOD, Dynamo, and Warp all contribute to your tilt series alignment, but the roles and contributions of each package have not been made clear. Automated alignment has supposedly been available in IMOD since 2017 (Mastronarde and Held JSB 197:102-113). At the very least, this paper should be cited, and the various approaches compared and contrasted. Describe why you think what you've done is an improvement over previous work.

Thank you for picking up on this, a citation has been added and some additional information is now provided in the manuscript. To elaborate a little further, we attempted to use IMOD but found that it failed to properly track fiducials for our data due to some assumptions in the tracking algorithm (some discussion here
https://groups.google.com/a/colorado.edu/g/imod/c/r1DFuEaIaZM/m/70YbxyAECAAJ)
As always, this is now probably fixed in IMOD. The big advantage of the Dynamo approach is in it's explicit attempts to reintegrate missing observations of fiducial positions and refine all positions against a per-fiducial reference, this leading to a larger number of fiducials which are well centered in the final model. IMOD recenters fiducials based on fiducial edges detected by sobel filtering. Our contribution here is simply to make the Dynamo approach more easily accessible for Warp users. We provide a wrapper with sensible defaults for automating tilt-series alignment, minimising required user input and dealing with all the metadata wrangling associated with getting alignments into Warp for subsequent tomogram reconstruction.

3) Line 149: Euler angle conversion routines are useful, but I question the benefit of yet another such package. Several are already available. E.g. in MATLAB the Aerospace Toolbox provides

euler2dcm and dcm2euler. In Python, packages scipy.spatial.transform and Pspincalc provide similar functionality. Moreover, in my opinion the fundamental issue is not Euler angle conversions per se, which are routine, but rather the lack of full documentation on conventions used by various EM packages. CCP-EM has attempted to summarize these, but their list is neither sufficiently complete nor always accurate, since it relies on the packages internal documentation. It would be extremely helfpul if someone would verify and summarizing complete rotation convention information for popular EM packages. Even assuming a right handed coordinate system, such a description must include:
A) Is the right-handed or left-handed screw rule used?
B) Are transformations active or passive?
C) Are Euler angles intrinsic or extrinsic?
D) Is the default transformatio reference-to-particle or vice-versa?
and, finally, of course,
E) The Euler or Tait-Bryan / Cardan sequence used.

We very much appreciate your valid concerns. Our limited experience of the MATLAB functions for dealing with rigid body rotations is that they do not provide enough information to be absolutely sure what is happening under the hood, see https://fr.mathworks.com/help/robotics/ref/eul2rotm.html as an example. We chose to provide a Python package as we believe the scientific community benefits from the ability to more easily share their work without worrying about licences. We are aware that it is possible to compile MATLAB into usable binaries which can run against the MCR (available freely), however this is a large dependency for such small utility packages.

Within the Python ecosystem, we were not aware of Pspincalc which appears powerful however lacks documentation and allows only for definition of the sequence of axes used (https://github.com/tuxcell/PSpincalc/blob/master/PSpincalc/PSpincalc.py) which, as you note, does not unambiguously define the transformation. An alternative can be found in Transforms3d (https://matthew-brett.github.io/transforms3d/) which is flexible and powerful but only deals with one transformation at a time, incurring the costs associated with large loops in python when dealing with large sets of Euler angles.

scipy.spatial.transform.Rotation is extreemely powerful but is not optimised for dealing with large sets of Euler angles. For a test case of 1 million Euler angles, we see a >10x speedup using the vectorised implementation in eulerangles rather than the scipy Rotation class (4.38s for scipy, 0.381s for eulerangles, benchmarking code : https://gist.github.com/alisterburt/4a32e9c122498ac0ab482ee5ba44ba10). In addition, the scipy class does not allow for specification of the rotation handedness.

Our package, in contrast, provides complete documentation https://eulerangles.readthedocs.io/en/latest/usage/quick_start.html, complete, unambiguous description of transformations in a right handed coordinate system and a mechanism for saving rotation conventions for future reuse. We completely agree that verification of the various conventions used and a summary of these data would be useful for the community – this could be provided in the form of a meta-package built on our package, with conventions described and easily reused (example, some conventions already defined in eulerangles https://github.com/alisterburt/eulerangles/blob/master/eulerangles/constants.py).

4) Lines 181-183: Why provide relion_star_downgrade, rather than updating Warp to (optionally) support the Relion 3.1 star format? Few users should still be using Relion 3.0, and you don't yourselves!

A valid point! Currently, Warp is not developed in the open as a project accepting external contributions, rather the source code is made available upon release of each new version (or at a later date when requested). Given that the developer stated in August 2020 that support for RELION 3.1 STAR files would be added in the next release (https://groups.google.com/g/warp-em/c/uAlXyZkCsbc/m/qR8idaUhCQAJ) we chose to provide this

script as a stop-gap for users in need (e.g. https://groups.google.com/g/warp-em/c/amJxqIkZOKY/m/DiUjgYpjCwAJ). As of 06/05/21 Warp still doesn't support RELION 3.1 STAR files.

5) Lines 198-199: Agreed, but many other packages already provide such exploratory data analysis capabilities. Dynamo, for example, already has access to a full range of EDA tools in MATLAB.

We are glad we agree. This is also our favourite aspect of working in Dynamo, however as above we think the open source nature of Python makes it a better choice for scientific tooling.

6) Lines 275-276: It's not strictly true that Relion uses the same mask for alignment, classification, and FSC's. During 3D refinement, Relion will use the same mask for alignment and FSCs, and also true that during 2D or 3D classification, if alignment is being done the same mask will be used for alignment and classification. However there is no requirement that the mask used during classification is the same as that during refinement or that either is the same as the mask used during final FSC computation or post-processing. Relion already provides most of the flexibility as you're describing.

We agree and have clarified this point in the text. Specifically, what is non-trivial to perform in RELION is a classification experiment in which, within each iteration, a larger masked region can be used for the alignment step and a smaller masked region is used for deciding which class a particle belongs to. This type of experiment has proven useful in our experience and although possible in RELION, is not provided in an easily accessible manner.

7) Figure 4: This figure and it's description could be improved. No offense, but your logos are hardly universally recognized. Many readers will not realize that a gear = Dynamo, a W = Warp, and M = M. Label them, add a legend, or make the description more complete.

This is a fair comment, the description has been updated accordingly.

8) Line 386: Rather than just "teamtomo.org", I suggest listing as a fully qualified url (and, of course, as a functional link in electronic versions).

Noted and changed, thank you.

Reviewer 3:
Summary of key results:
This paper provides a comprehensive workflow for solving structures using subtomogram averaging from cryo-electron tomography data. The authors build upon algorithmic approaches already established in the field, and develop a series of scripts to automate and facilitate the process of interacting between various programs. The end result is a computational framework to make it easier to process tilt series, reconstruct tomograms, pick particles, and incorporate a priori orientation information calculated from multiple sources to facilitate ab initio subtomogram averaging. This workflow is benchmarked on two datasets of membrane-associated protein complexes, the HIV-1 CA-SP1 hexamer and chemosensory arrays, yielding results both comparable to previous reports and with some additional novel structural insights. The authors also establish an open-access and collaborative platform to disseminate their pipeline and to encourage other tomographers to contribute their respective protocols outlining various steps of the cellular tomography workflow.

Strengths, originality, and significance:
It is often challenging for newcomers, particularly those lacking extensive scripting and programming skills, to navigate through the tomography reconstruction and subtomogram averaging pipeline to produce a high resolution and/or biologically-interpretable structures. Although there have been some efforts in recent years by groups to alleviate this steep learning curve, these workflows are often restricted to a single software package without much flexibility to

navigate and utilize beneficial aspects of multiple algorithmic approaches. If we want to expand the user-base of cryo-electron tomography to both structural and cellular biologists alike, we need to develop more streamlined and user-friendly workflows. This manuscript explicitly addresses this need and opens up an exciting option for the collective tomography community to take part in this effort through an open-access online platform.

Suggested improvements:
Overall, the content of the manuscript is excellent and the streamlined approach is very much needed in the field. However, the overall format, organization, and presentation of the manuscript currently comes across a bit clunky and non-linear. It tends to jump around between different steps, providing details in some sections, and then again more details in other sections (e.g. explanation of Euler angles, mention of scripts in both the methods and results/discussions section, etc.). The manuscript would be greatly improved through restructuring and reorganization, with the goal in mind of it being used primarily (and in practice) as a protocol for subtomogram averaging. One suggestion would be to restructure the manuscript with the target audience in mind, which is most likely a user that has either (1) just started their structure-solving journey (with little to no experience) or (2) tried using a single program and was left frustrated that initial attempts failed to produce an interpretable structure. In both cases, the audience likely has some knowledge of the process, but are searching for a "how-to" manual that will guide them through the (often confusing) process.

Here are a few actionable suggestions:

1. Reformat manuscript to have more of a protocol format. Perhaps sections for the manuscript could include the following: Introduction, Overview of approach, application of methodology, Conclusion.

Your comments are very much appreciated and we identify with your reaction. Indeed, one of the first incarnations of this manuscript was, as suggested, a ‘protocol/how-to manual’. We quickly found that it was extremely difficult to simultaneously provide the high-level overview of our approach, the motivation for such an approach and enough detail in the procedural how-to sections whilst adequately explaining our specific contribution.

Instead, we opted to explicitly divide the work into two parts. This manuscript, explaining our contribution (the tools we provide), a high level overview of the framework they enable and illustrated examples of working within this framework for two real-world datasets. We chose to then provide the procedural/how-to guide separately as https://teamtomo.org/walkthroughs/EMPIAR-10164/introduction.html. The benefits of providing this resource external to the manuscript as a web resource rather than a single, linear document are twofold :

1) The ability to go into as much detail as is necessary without breaking the flow of the procedural guide. E.g. In the initial model generation section of the guide (https://teamtomo.org/walkthroughs/EMPIAR-10164/ini-model.html#aligning-the-particles) we provide a fairly minimal, specific example of the project setup for deriving an initial model from the HIV data. We also link to a more detailed mini-tutorial (https://teamtomo.org/mini-tutorials/dynamo/simple-alignment.html) which provides all the detail necessary to set up and run such an alignment experiment.
2) The ability to easily update the guide to reflect inevitable changes in the software ecosystem. We recognise that the software ecosystem is changing around us and don’t think that we can provide, in good conscience, a fixed document which will remain applicable given that features like automated tilt-series alignment should soon be available in Warp.

To address your concerns, we have placed much more emphasis on the existence of the how-to guide in the manuscript and made an effort to more clearly convey the purpose of this manuscript. We hope you find these modifications satisfactory.

2. The introduction generally reads a bit clunky and does not build enough rationale for the described workflow. As someone who has experienced firsthand the frustration and annoyance of navigating through the various software packages, the rationale is very obvious to me, and is why I believe the content of this study is particularly important and relevant for the field. However, I worry that without this "insider" knowledge, the importance of this work might be missed. Perhaps better to build rational by listing all of the various steps needed for tomogram reconstruction and subtomogram averaging, and give examples to illustrate why it is difficult to integrate and interact with all of them (e.g. different file inputs and outputs, etc.). For example, lines 258-279 are great rationale and would be more appreciated in the introduction.

We have restructured the introduction to highlight the rationale behind the approach to data processing we enable and hope this satisfies your request.

3. "Overview of approach" could contain sub-sections that go over various steps, in order. Content-wise, this would include everything from "materials and methods" through "framework for flexible ab initio...". It would be better to re-format as a protocol for the workflow, with links to the various scripts embedded in the text. Lines 299-324 read more like a methods section, and it would be useful to include in the "overview of approach" or perhaps even in a supplemental methods section.

We believe that this is adequately addressed by the existence of the complete guide to processing the HIV dataset EMPIAR-10164
https://teamtomo.org/walkthroughs/EMPIAR-10164/introduction.html

4. It would be helpful to adapt figure 4 to make it explicit which scripts are novel to this work, and which are already established. Some of the scripts are shown, but not all of them, and it gets a bit lost which ones were developed in this study.

We thank you for this suggestion, we have modified the figure legend to better highlight our contributions.
